# There is an obstetrical dilemma: Misconceptions about the evolution of human childbirth and pelvic form

**DOI:** 10.1002/ajpa.24802

**Published:** 2023-06-23

**Authors:** N. D. S. Grunstra, L. Betti, B. Fischer, M. Haeusler, M. Pavlicev, E. Stansfield, W. Trevathan, N. M. Webb, J. C. K. Wells, K. R. Rosenberg, P. Mitteroecker

**Affiliations:** ^1^ Department of Evolutionary Biology, Unit for Theoretical Biology University of Vienna Vienna Austria; ^2^ Mammal Collection Natural History Museum Vienna Vienna Austria; ^3^ School of Life and Health Sciences University of Roehampton London UK; ^4^ Institute of Evolutionary Medicine University of Zurich Zurich Switzerland; ^5^ School for Advanced Research Santa Fe New Mexico USA; ^6^ Institute of Archaeological Sciences, Senckenberg Centre for Human Evolution and Palaeoenvironment Eberhard‐Karls University of Tübingen Tübingen Germany; ^7^ UCL Great Ormond Street Institute of Child Health, Population, Policy and Practice Research and Teaching Programme Childhood Nutrition Research Centre London UK; ^8^ Department of Anthropology University of Delaware Newark Delaware USA

**Keywords:** antagonistic selection, bio‐cultural evolution, bipedal locomotion, human childbirth, obstructed labor, pelvis

## Abstract

Compared to other primates, modern humans face high rates of maternal and neonatal morbidity and mortality during childbirth. Since the early 20th century, this “difficulty” of human parturition has prompted numerous evolutionary explanations, typically assuming antagonistic selective forces acting on maternal and fetal traits, which has been termed the “obstetrical dilemma.” Recently, there has been a growing tendency among some anthropologists to question the difficulty of human childbirth and its evolutionary origin in an antagonistic selective regime. Partly, this stems from the motivation to combat increasing pathologization and overmedicalization of childbirth in industrialized countries. Some authors have argued that there is no obstetrical dilemma at all, and that the difficulty of childbirth mainly results from modern lifestyles and inappropriate and patriarchal obstetric practices. The failure of some studies to identify biomechanical and metabolic constraints on pelvic dimensions is sometimes interpreted as empirical support for discarding an obstetrical dilemma. Here we explain why these points are important but do not invalidate evolutionary explanations of human childbirth. We present robust empirical evidence and solid evolutionary theory supporting an obstetrical dilemma, yet one that is much more complex than originally conceived in the 20th century. We argue that evolutionary research does not hinder appropriate midwifery and obstetric care, nor does it promote negative views of female bodies. Understanding the evolutionary entanglement of biological and sociocultural factors underlying human childbirth can help us to understand individual variation in the risk factors of obstructed labor, and thus can contribute to more individualized maternal care.

## 1 THE OBSTETRICAL DILEMMA

The evolution of human childbirth and pelvic morphology has been an intensively investigated and debated topic in recent years. Most researchers have considered the tight fetopelvic fit and relatively risky human childbirth as the result of multiple, partly opposed selective factors (e.g., Grunstra et al., 2019; Haeusler et al., 2021; Krogman, 1951; Pavlicev et al., 2020; Rosenberg & Trevathan, 1996, 2021; Wall‐Scheffler et al., 2020; Washburn, 1960; Wells et al., 2012). Washburn (1960) coined the term “obstetrical dilemma” to describe the functional and evolutionary conflict between a spacious birth canal and a small pelvis for bipedal locomotion, and suggested that the relative immaturity of human newborns is an evolutionary “solution” to this dilemma (see Dunsworth, 2021 and Haeusler et al., 2021 for the different usages and the history of the term “obstetrical dilemma”). Apart from upright locomotor performance, other selective factors, such as pelvic floor stability and thermoregulation, have likely affected the evolution of the human pelvis: an obstetrical dilemma in a wider sense. In addition to these evolutionary, population‐level dynamics, individual fetal growth and maternal pelvic dimensions are influenced by numerous environmental and lifestyle factors that have been subject to continual change throughout the human past and can increase the risk of obstructed labor (“developmental plasticity,” e.g., Wells, 2015, 2017; Zaffarini & Mitteroecker, 2019). However, there has been a growing tendency among some anthropologists and human biologists to downplay the “difficulty” of human childbirth due to tight fetopelvic relationships and to reject the evolutionary origin of this situation in multiple, partly antagonistic selective forces. In part, this stems from the motivation to combat the increasing pathologization and overmedicalization of childbirth in some industrialized countries and to draw attention to sociocultural, economic, and political factors underlying global maternal and neonatal mortality. Several authors have argued that there is no obstetrical dilemma at all, and emphasized that the difficulty of childbirth in modern societies is amplified by inappropriate obstetric practices, such as disadvantageous birth positions, patriarchal cultural and medical systems, as well as lifestyles that increase the risk of obstructed labor (e.g., Dunsworth, 2018; Gorman et al., 2022; Stone, 2016; Walrath, 2017). The fact that lifestyle and obstetric practices indeed affect parturition, along with the failure of some studies to identify biomechanical and metabolic constraints on pelvic dimensions (Vidal‐Cordasco et al., 2017; Warrener et al., 2015), has sometimes been interpreted as empirical support for discarding evolutionary explanations altogether. We fully sympathize with the motivation to de‐pathologize human birth. However, in this short paper, we explain why these important points do not invalidate the idea of an obstetrical dilemma in which pelvic and fetal dimensions evolved in response to multiple opposed selective forces that have varied in magnitude throughout human evolution and that still vary geographically. We show that there is robust empirical evidence and solid evolutionary theory supporting the notion of an obstetrical dilemma in the wider sense.

## 2 HUMAN CHILDBIRTH IS NOT PATHOLOGICAL, BUT THE FETOPELVIC FIT IS GENERALLY TIGHTER, AND HENCE BIRTH IS RISKIER, THAN IN MOST OTHER PRIMATES

Maternal and neonatal mortality and morbidity rates increase dramatically in the absence of midwifery and medical intervention, with obstructed labor and its sequelae resulting from fetopelvic disproportion being major contributors across different temporal, cultural and geographical contexts (Ayenew, 2021; GBD, 2015; Haeusler et al., 2021; Khan et al., 2006; Prual et al., 2000; Say et al., 2014; Wall, 1998; Wall, 2006). Midwifery is widespread culturally and has likely been a critical part of our bio‐cultural evolution by improving birth outcomes (Mitteroecker & Fischer, 2023; Rosenberg & Trevathan, 2001; Trevathan, 1987, 1988). Actively assisted birth appears to be very rare in other primates; routine assistance during birth is ubiquitous in and unique to humans. The age‐old practice and tangible benefits of midwifery indicate that human childbirth is difficult and riskier without it.

**FIGURE 1:**
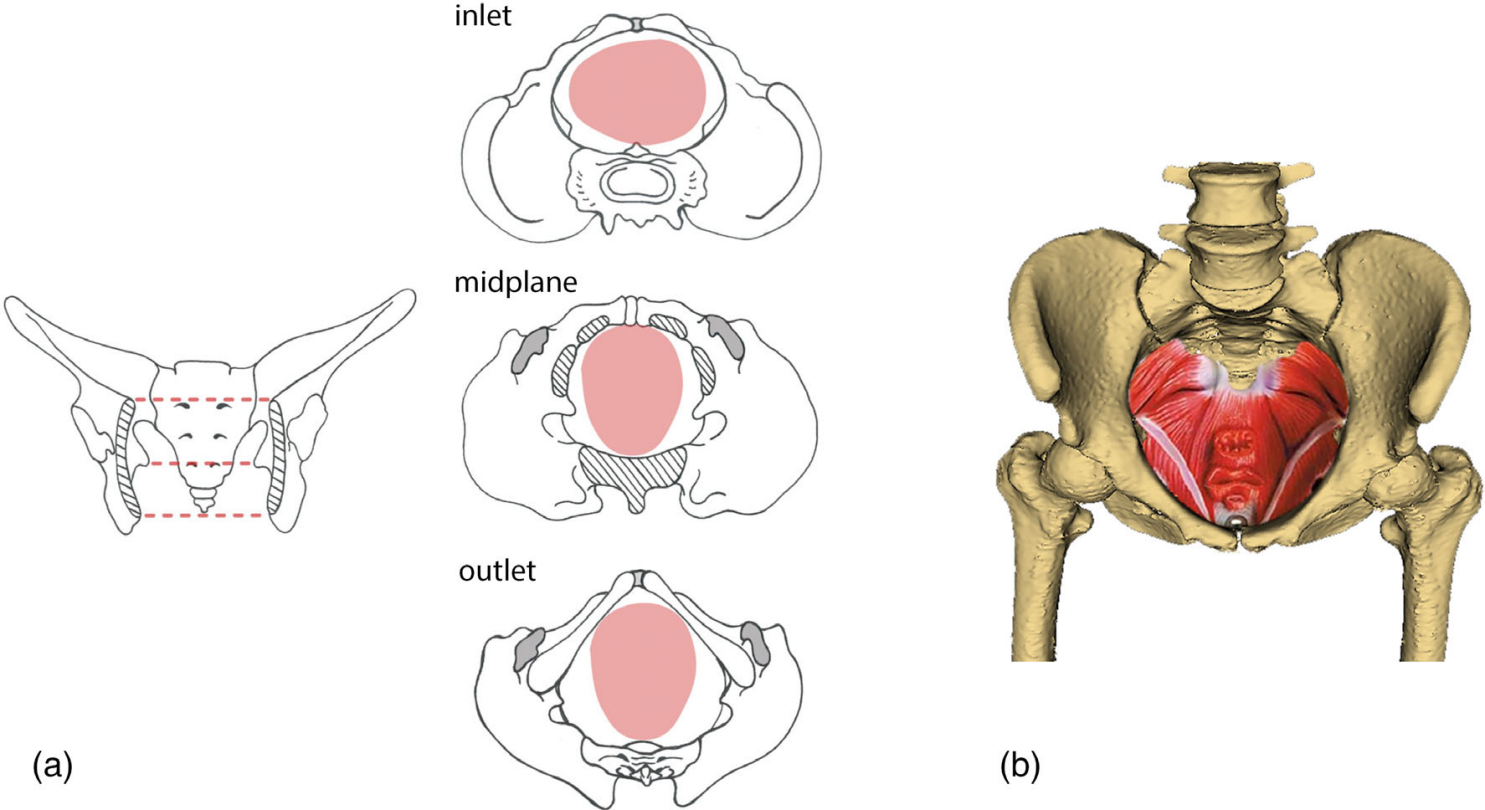
(a) Schematic illustration of the tight fetopelvic fit typical for humans in the different pelvic planes (from top to bottom): inlet, midplane, and outlet. The midplane and outlet are a “midwife's view” of the fetus as it passes through the birth canal. In order for the maximum dimensions of the fetal head to align with the maximum dimensions of the birth canal and the pelvic floor, fetal rotation through the different planes is typical, although individual variation exists in the exact orientation and flexion of the fetal head. The mother's pubic symphysis is at the top of the drawing and her sacrum at the bottom. The fetal head is shaded pink. (b) Anterior‐cranial view of the human bony pelvis, with female pelvic floor muscles, ligaments and connective tissue superimposed inside the pelvic canal. Anterior‐posteriorly, the urethral, vaginal, and rectal openings are visible. The left and right femora are articulated to the bony pelvis to form the hip joints.

Clearly, most women are able to give birth vaginally when receiving adequate midwifery care; fetopelvic disproportion affects only a small percentage of all births. However, it is indubitable that a relatively larger or more flexible birth canal, as found in many other primates, would generally ease human childbirth and reduce injuries to both mother and baby. (This is not in contradiction with the fact that, in modern obstetrics and midwifery, other individual risk factors are equally or more important than anatomical limitations.) A tight fetopelvic fit characterizes modern humans as a species, which usually necessitates a complex pattern of fetal rotation, head flexion and extension during birth (Figure 1a). Some kind of fetal rotation has also been observed in several non‐human primates (Hirata et al., 2011; Stoller, 1995), and the fetopelvic fit may also be tighter in apes than previously assumed (Laudicina & Cartmill, 2023; Webb et al., 2021), but the human combination of a very tight fetopelvic fit, relatively immobile pelvic joints, and rotational birth appears to be unique among primates. The observations from non‐human primates do not disprove the difficulty of human childbirth and its evolutionary history; they just show that a tight fetopelvic fit extends, to variable degrees, far beyond modern humans (e.g., Fischer et al., 2021; Frémondière et al., 2022; Schultz, 1949; Wells et al., 2012).

## 3 THERE IS STRONG EMPIRICAL EVIDENCE OF FUNCTIONAL TRADEOFFS AND ANTAGONISTIC SELECTION IN THE HUMAN PELVIS

Natural selection arises from the association between a trait and fitness (average number of offspring associated with a trait value). Without experimental evidence, it is difficult to document natural selection and even more so to demonstrate that selection has caused an evolutionary change. For this reason, adaptation has sometimes been invoked too readily as an explanation in evolutionary biology and anthropology (Gould & Lewontin, 1979; Orzack et al., 2001). However, for questions on human evolution, modern medical, epidemiological, and biomechanical data provide insights into natural selection, even though it may not be straightforward to infer past selective regimes from modern health data. For instance, some conditions, such as pelvic floor disorders, that often are highly inconvenient today, were probably severely detrimental or even lethal in the past and still are so in many low‐income countries (Pereira et al., 2020; Walker & Gunasekera, 2011; Wall, 1999). Other conditions, such as macrosomic fetuses, that hinder vaginal delivery today may have been very rare in our pre‐agricultural past due to a lower‐caloric diet (Wells et al., 2012). Medical data can also be insufficient to make inferences about longer evolutionary time scales because modern human variation may no longer contain phenotypes that were selected against in the past.

Nonetheless, there is overwhelming evidence of selective forces related to human childbirth. Medical data document an increased risk of obstructed labor in mothers with a smaller birth canal, which inevitably imposes natural selection for a more spacious pelvic canal (“obstetric selection”) (e.g., Adadevoh et al., 1989; Daghighi et al., 2013; Franz et al., 2017; Korhonen et al., 2015; Starrach et al., 2023; Zaretsky et al., 2005). Modern obstetrics has relaxed this selective pressure in high‐income countries, but maternal and neonatal mortality and morbidity resulting from obstructed labor are still high in regions with insufficient access to healthcare. Conversely, medical and biomechanical studies indicate that a wider or more spacious birth canal increases the risk of pelvic floor disorders (Figure 1b), such as pelvic organ prolapse and incontinence (Handa et al., 2003; Stansfield et al., 2021; Stav et al., 2007; Sze et al., 1999), which negatively affect the chances of survival and reproduction (Rogers et al., 2018; Wall, 1999). Pelvic floor disorders have a complex etiology and pelvic dimensions are only a minor risk factor. Nonetheless, their association with pelvic canal size, even in younger and nulliparous women (e.g., Hendrix et al., 2002; Lawrence et al., 2008; Nygaard et al., 2008), imposes natural selection toward a smaller birth canal. In addition, not only the maternal pelvis but also the fetal head and shoulders are subject to obstetric selection favoring a smaller fetus (Kawada et al., 2022). Simultaneously, however, smaller fetal size at birth reduces infant survival rate and thus is selected against (Alberman, 1991). All these factors create a complex landscape of antagonistic selective forces acting on both maternal and fetal dimensions.

Since the beginning of the 20th century, bipedal locomotion has been widely accepted as *the* source of selection opposing obstetric selection, but in the last decade this idea has become increasingly controversial (e.g., Dunsworth et al., 2012), although few studies have actually tested this hypothesis empirically. Warrener et al. (2015) detected no correlation of locomotor cost with hip width in a small sample of US male and female college students, although hip abductor costs were significantly higher and mechanical advantage significantly lower in women compared to men. Vidal‐Cordasco et al. (2017) similarly detected slightly higher, albeit statistically non‐significant, mass‐adjusted walking costs in their sample of Spanish women compared to men. Moreover, greater pelvic breadth in females has been shown to yield additional speed flexibility and greater stride length, partly compensating for elevated locomotor costs owing to lower hip mechanical advantage (Gruss et al., 2017; Rak, 1991; Wall‐Scheffler, 2022; Wall‐Scheffler & Myers, 2017; Whitcome et al., 2017). These results led several authors to reject the hypothesis that efficient bipedal locomotion has restricted the evolution of a more spacious birth canal (e.g., Dunsworth, 2016, 2018; Warrener et al., 2015, Warrener, 2018; Gorman et al., 2022). Some authors questioned (Nowell & Kurki, 2020; Warrener et al., 2015) or even rejected (e.g., Dunsworth, 2018, 2021; Gorman et al., 2022) the entire notion of an evolutionary tradeoff subsumed under the classic obstetrical dilemma hypothesis.

**FIGURE 2:**
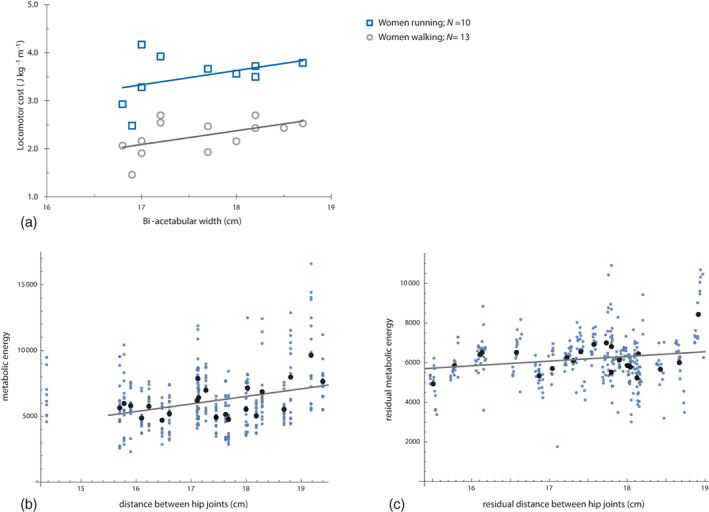
(a) Reanalysis of the Warrener et al. (2015) data by Haeusler et al. (2021), showing that locomotor costs of walking and running weakly increase with pelvic width in women. (b) Reanalysis of the data by Kramer and Sylvester (2023), showing metabolic energy expenditure of the abductor muscles versus hip width for 348 trials of 23 women in a musculoskeletal model (blue points). The black points are averages over all trials for each woman. These data also demonstrate an increase of energy expenditure with pelvic width (*p* < 0.001, excluding the outlier with a very small pelvis). (c) When correcting for body mass, stature, and velocity, the slope drops to about a third but remains significant (*p* = 0.017, based on a linear mixed effects model with type III sum of squares that allows for random slope and intercept of velocity for each individual).

We believe that this conclusion is premature. In fact, in a reanalysis of the data of Warrener et al. (2015), Haeusler et al. (2021) found energy consumption during walking and running to increase weakly with bi‐acetabular width in women. Using a musculoskeletal model of the human body, Kramer and Sylvester (2023) reported that the distance between the hip joints (“hip width”) does not predict metabolic energy expenditure of the abductor muscles. However, in a reanalysis of their data, we found that hip width does correlate with energy expenditure, weakly but significantly even if body mass, stature, and velocity are statistically controlled for (Figure 2). Moreover, biomechanical theory clearly predicts such an effect (Lovejoy et al., 1973; Ruff, 1995) despite other factors contributing to locomotor efficiency as well. Recent studies that failed to detect this effect are small and do point in the direction expected under the bipedalism hypothesis (see also Ruff, 2017). However, given that bipedalism evolved at least 4 million years ago and that the sample in the Warrener et al. (2015) study consisted of young, recreational runners, one would only expect a very weak, if any, association between pelvic form and locomotor performance. Individuals with a pelvic geometry clearly disadvantageous for running were likely “selected out” throughout evolution and, to some degree, also by study design through the inclusion of recreational runners only in the Warrener et al. (2015) study (as pointed out also by Kramer & Sylvester, 2023).

Several studies of the obstetrical dilemma focused on the sole comparison of male and female trait means—without reference to individual variation—but this can be highly misleading. Biomechanical theory predicts that larger hip width increases locomotor costs, all else being constant. If true, this would manifest as an association between pelvic form and locomotor costs across individuals, independent of variation in other traits. In a sufficiently large sample of individuals, the different effects on locomotor costs can be disentangled statistically, but when comparing only two sex means it is impossible to distinguish the effects of pelvic morphology from other dimorphic morphological, physiological, and behavioral characteristics that also affect biomechanics (e.g., gait pattern or infant‐carrying; Wall‐Scheffler & Myers, 2017; Wall‐Scheffler, 2022). Sex differences or similarities in locomotor costs therefore do not allow for any direct inference about their biomechanical or behavioral cause. Similar limitations apply to the comparison of extinct species: one cannot infer walking efficiency in australopithecines just from their pelvis, which is much wider than in modern humans. An association between pelvic dimensions and locomotor efficiency across individuals suffices to impose natural selection, but different species and also different sexes might show different responses to a shared selection pressure.

Additional research is necessary, ideally across a larger range of individual variation in body form, to gauge the relevance of the bipedalism hypothesis as one piece of a certainly much more complex evolutionary scenario. It is important to also consider locomotor cost in terms of energy expenditure as well as the risk of injury (Ruff, 2017) and other aspects of upright bipedality (e.g., balance and load carrying; Wall‐Scheffler, 2022), and not only pelvic width but also antero‐posterior depth (Haeusler et al., 2021; Stansfield, Fischer, et al., 2021) and the whole pelvic‐femur complex (Ruff, 2017). Moreover, locomotor efficiency should be considered across a wide range of types, including walking, sprinting, and long‐distance running (Kramer & Sylvester, 2023).

The specific antagonistic selective regime explains the astonishing persistence, though to variable degrees, of cephalopelvic disproportion throughout human evolution. Selection for a small birth canal is likely relatively weak and approximately linear (reviewed in Mitteroecker et al., 2016). For instance, the risk of pelvic floor disorders increases roughly linearly with pelvic canal size. However, selection for a sufficiently large pelvic canal is truncational: if the birth canal is not large enough for the fetus, fitness drops sharply. As a result, the phenotype distribution with maximal population fitness necessarily involves individuals with too narrow a birth canal (Mitteroecker et al., 2016). This argument is built on a model, not on direct empirical observations, but the underlying theory is well established and successfully explains the evolutionary dynamics and heritability of many other traits and diseases (Mitteroecker, 2019; Mitteroecker et al., 2017; Urban et al., 2013).

## 4 THE PRONOUNCED SEX DIFFERENCES IN THE HUMAN PELVIS ATTEST TO THE SEX‐SPECIFIC SELECTIVE HISTORY

Females tend to have absolutely (not just relatively) larger pelvic canal dimensions in all human populations studied to date. Pelvic sex differences are not a simple corollary of differential overall growth: they are opposite to the pattern of dimorphism in overall body size and cannot be explained by allometric scaling alone (Arsuaga & Carretero, 1994; Fischer & Mitteroecker, 2017; Kurki, 2011; Wood & Chamberlain, 1986). Instead, they must be the result of sex differences in the tradeoff among the selective factors on the pelvis (reviewed in Grunstra et al., 2019, Haeusler et al., 2021 Pavlicev et al., 2020). A wealth of empirical studies documents that among primates and other placental mammals, the magnitude of pelvic sex differences is positively correlated with relative neonatal size, a reasonable proxy for the strength of obstetric selection (Fischer et al., 2021; Grunstra et al., 2019; Leutenegger, 1974; Moffett, 2017; Ridley, 1995; Schultz, 1949; Tague, 2005; Zollikofer et al., 2017), with humans exhibiting some of the most pronounced pelvic dimorphism among primates (Moffett, 2017; Tague, 2005).

Dunsworth (2020, 2021) has questioned the importance of genetic factors in the sex‐specific development of the human pelvis and criticized the interpretation of pelvic dimorphism as an adaptation to give birth to large human neonates. She suggested that the size of the female pelvic organs together with higher female levels of estrogen and relaxin induce a larger pelvic canal. These developmental pathways may or may not contribute to the sex‐specific development of the human pelvis, but either way they are not in contradiction with an evolved genetic basis. Most aspects of bone growth and development are controlled via hormonal and mechanical stimuli jointly. However, the expression of these hormones and the level of response to the hormonal and mechanical cues are, at least partly, under genetic control and differ, on average, between the sexes. For instance, sex differences in estrogen and relaxin receptors seem to be present in the fetal pelvis already (Maclennan & Maclennan, 1997; Dehghan et al., 2014; Loder & Skopelja, 2011). In population genetics, “genetic control” merely implies a correlation of a phenotypic value with alleles at one or more genetic loci. All effects of genetic loci are necessarily mediated via developmental pathways (such as through hormonal action). The relatively high heritability of pelvic dimensions (Sharma, 2002) along with a considerable global variation in the magnitude (but not in the pattern) of pelvic dimorphism (Betti, 2014; DelPrete, 2019; Fischer et al., 2021; Kurki, 2011, 2013) indicates such genetic control of pelvic form variation, which is sufficient for natural selection to act on at a microevolutionary level.

## 5 GLOBAL VARIATION IN PELVIC DIMENSIONS IS PARTLY THE RESULT OF NATURAL SELECTION

Pelvic dimensions show considerable global variation across recent and historic human populations (e.g., Betti & Manica, 2018; DelPrete, 2019; Kurki, 2013). A related debate is whether these differences primarily originated from “neutral evolution” (genetic drift), phenotypic plasticity, or local evolutionary adaptations. Genetic drift always occurs, even in the presence of strong selection on multiple traits, and some aspects of pelvic variation may indeed have resulted from drift. However, the magnitude of the population differences in pelvic form clearly exceeds the magnitude expected under a model of purely neutral evolution (Mitteroecker et al., 2021). Moreover, some aspects of pelvic differences in the breadth of the upper pelvis as well as in the pelvic canal correlate globally with environmental temperature, as expected for thermoregulatory adaptation (e.g., Betti et al., 2014; Betti & Manica, 2018; Cho et al., 2022; Kurki, 2013; Mitteroecker et al., 2021; Ruff, 1994, 2002). Global variation in environmental and sociocultural factors, and thus the magnitude of the various selective forces, likely has led to different local evolutionary “compromises” and contributed to the variation in pelvic form and the difficulty of labor visible today (Wells, 2015).

## 6 HUMAN GESTATION LENGTH IS NOT TRUNCATED, BUT THIS DOES NOT CONTRADICT THE NOTION OF A “DILEMMA”

Humans have often been touted as being born “early” that is, following a comparatively short gestation length that protects childbirth from the pelvic constraints imposed by bipedalism, which has been argued to explain the relative neurological immaturity and “helplessness” of human neonates (Gould, 1977; Portmann, 1941; Washburn, 1960). This view has been prone to misinterpretation. At birth, human newborns possess no more than 30% of adult brain mass, compared to 40%–65% in the great apes (Barton & Capellini, 2011; DeSilva & Lesnik, 2006). However, humans have a gestation length expected for a primate of our body size, close to what we see in great apes, as well as neonatal body and brain sizes expected—or even higher than expected—for a female primate of our size (Dunsworth et al., 2012; Martin, 2007; Rosenberg, 1992; Wells et al., 2012). Presumably, multiple factors have influenced the evolution of human gestation length, including the necessity to fit the fetus through the birth canal. Dunsworth et al. (2012) suggested that maternal energy resources (a metabolic cap) trigger the end of pregnancy, not pelvic constraints, and that this is an alternative explanation to the “obstetrical dilemma” for the timing of birth and, indirectly, for neonatal size (but see Cordey et al., 2022 and Haeusler et al., 2021). This is an important perspective, but fetal size, maternal metabolic investment, and pelvic canal size very likely have coevolved because they all need to fit. Whatever the proximate triggers of parturition and determinants of gestation length are (which differ among species and are still not understood in humans), this does not refute a scenario of antagonistic selection in the human pelvis, which is documented by medical data and remains the most powerful explanation of the observed magnitude and pervasive pattern of pelvic sex differences. The “dilemma” in terms of a functional tradeoff in the human pelvis is still real, even if the once‐proposed “solution” of early birth in terms of gestation length is not (see Haeusler et al., 2021).

## 7 ENVIRONMENTAL, CULTURAL, AND LIFESTYLE FACTORS CAN AFFECT FETO‐PELVIC RELATIONSHIPS AND THE DIFFICULTY OF LABOR

As for basically any other phenotypic trait, fetal size and the form of the maternal birth canal are not only genetically determined. Many environmental, nutritional, and lifestyle factors influence the individual development of these traits and may disrupt the close match in size between the fetus and the maternal pelvis. Environmental and sociocultural transitions can affect—and repeatedly have affected—fetopelvic relationships and the underlying evolutionary equilibrium. In particular, life‐course exposure to the double burden of malnutrition, through undernutrition in early life followed by the onset of overweight, often leads to stunted growth and a reduced capacity of the birth canal, which entails an increased risk of obstructed labor (Wells, 2015, 2017; Wells et al., 2018). Consequently, rapidly improving living conditions can increase the risk of fetopelvic disproportion because they lead to accelerated fetal growth while maternal dimensions lag behind one generation (Wells, 2017; Zaffarini & Mitteroecker, 2019). Advancements in midwifery and obstetric care can affect the success of labor, independently of biological factors.

All these environmental, cultural, and lifestyle factors have repeatedly changed—often but not always for the better—during human evolution up to the present, yielding a variable and “contemporary obstetrical dilemma” (Wells, 2017; Wells et al., 2012). They have affected maternal and fetal growth and the success of labor by ameliorating or exacerbating the fetopelvic fit. The growing body of literature on cultural practices and lifestyle differences that mediate birth outcomes demonstrates the rather precarious balance between maternal and fetal dimensions. However, these transitions have only modified but not fundamentally changed or even stopped the evolutionary dynamics.

## 8 THERE IS AN OBSTETRICAL DILEMMA, AND EVOLUTIONARY EXPLANATIONS NEITHER PATHOLOGIZE CHILDBIRTH NOR HINDER MIDWIFERY CARE

Describing the evolutionary compromise underlying the human birth canal does not logically imply that women are “compromised” as if they were “weakened” or “flawed” (as implied by Dunsworth, 2018, 2021, and explicitly asserted by Stone, 2016 and Walrath, 2003, 2017), nor does it pathologize childbirth in humans or justify unwarranted medical interventions. We consider this an important distinction. Humans are unique in having large neonates and being fully bipedal, but like many other traits, the pelvis likely is subject to antagonistic selection to variable degrees in all primates and other amniotes. Evolutionary compromises in complex organisms are the rule rather than the exception (e.g., Ghalambor et al., 2003; Haig, 1993; Hill & Kaplan, 1999; Mitteroecker, 2019; Polly, 2020; Wells, 2003); they are not confined to female human bodies. A comparative view clearly suggests that maternal and fetal dimensions in humans have been subject to similar evolutionary forces as in other amniotes. Like in all sciences of the past, an evolutionary explanation cannot be definitively “proven,” but the medical, epidemiological, biomechanical, and comparative data strongly support an antagonistic selective regime underlying the human birth canal: an obstetrical dilemma, yet much more complex than originally conceived by Washburn and others in the 20th century. We are convinced that research on this evolutionary history need not hinder appropriate and evidence‐based midwifery and obstetric care today, nor does it promote negative views of female bodies and women's capabilities. To the contrary, we believe that understanding the evolutionary entanglement of biological and sociocultural factors underlying human childbirth helps us to understand individual and global variation in the risk factors of obstructed labor, and thus can contribute to more individualized and less‐invasive maternal care.

## AUTHOR CONTRIBUTIONS

**L. Betti:** Investigation (equal); writing – review and editing (equal). **B. Fischer:** Investigation (equal); writing – review and editing (equal). **M. Haeusler:** Investigation (equal); writing – review and editing (equal). **M. Pavlicev:** Investigation (equal); writing – review and editing (equal). **E. Stansfield:** Investigation (equal); writing – review and editing (equal). **W. Trevathan:** Investigation (equal); writing – review and editing (equal). **N. M. Webb:** Investigation (equal); writing – review and editing (equal). **J. C. K. Wells:** Investigation (equal); writing – review and editing (equal). **K. R. Rosenberg:** Conceptualization (equal); investigation (equal); writing – original draft (equal); writing – review and editing (equal). **P. Mitteroecker:** Conceptualization (equal); investigation (equal); methodology (equal); writing – original draft (lead); writing – review and editing (equal). **N. D. S. Grunstra:** Conceptualization (equal); investigation (equal); writing – original draft (lead); writing – review and editing (equal).

## CONFLICT OF INTEREST STATEMENT

The authors declare no conflict of interest.
